# Comparison of nasal patency after nose-blowing between pinch versus no pinch method: a randomized controlled trial

**DOI:** 10.1038/s41598-021-01631-6

**Published:** 2021-11-11

**Authors:** Patorn Piromchai, Jakkrit Netnoi, Supaporn Srirompotong, Panida Thanawirattananit

**Affiliations:** Department of Otorhinolaryngology, Faculty of Medicine, Khon Kaen University, Khon Kaen, 40002 Thailand

**Keywords:** Respiratory tract diseases, Infectious diseases

## Abstract

We proposed that nose-blowing without pinching was safer and able to get rid of mucus and maintain nasal patency as effective as the pinch and blow method. The objective of this study was to evaluate the nasal patency after nose-blowing by pinching the nose versus no pinching. The patients who have nasal discharge such as allergic rhinitis or common cold were recruited. The patients were randomized to perform pinching or no pinching nose-blowing. Fifty patients were enrolled in this study. The objective evaluation using acoustic rhinometry found no difference in nasal patency between the two groups (p > 0.05). The subjective patency score was significantly higher in the pinch one nostril shut group (mean difference 0.88, 95% CI 0.20–1.55). The patency of the two methods were comparable according to the objective test. However, the patients felt that their nose was clearer when pinching and blowing.

## Introduction

A respiratory infection can be transmitted through droplets or aerosol particles^1^. Breathing, talking, or coughing are capable to be a source of respirable particles carrying infectious virus^2^. Bioaerosol transmission usually occurs when a person is in close contact with a respiratory disease patient created by coughing, sneezing or other aerosol-generating procedures such as nose-blowing^3,4^.

Nose-blowing is the act to get rid of nasal mucus by forcefully exhale through the nose. The handkerchief or facial tissues were usually used to cover the nose, prevent the mucus or droplet spreading and remove the mucus remnant from the nose^5^. This procedure can be done by pinching the nose to generate more exhalation pressure or without pinching.

The pressure generated during nose-blowing with pinching is much higher than the pressures generated during nose-blowing without pinching. One study found the pressure during nose-blowing was around 500 daPa without pinching but increased to around 900 daPa when the nostril was blocked^6^.

Moreover, forceful nose-blowing can cause adverse events such as epistaxis or mucosal injury. The adverse events can be severe such as pneumocephalus^7^, pneumolabyrinth^8^, and orbital blowout fracture^9^. Additionally, the nasal fluid can be propelled into the paranasal sinus or middle ear causing rhinosinusitis or otitis media^10^.

We proposed that nose-blowing without pinching is safer and able to get rid of mucus and maintain nasal patency as effective as the pinching and blowing method. This study's objectives were to evaluate the nasal patency and middle ear pressure after nose-blowing by pinching versus no pinching.

## Methods

### Study design and setting

This randomized controlled trial was conducted from September 2018 to March 2021 at Khon Kaen University, Faculty of Medicine, Department of Otorhinolaryngology, Thailand.

### Participants

The patients aged more than 20 years who have nasal mucus such as allergic rhinitis or common cold were invited to participate in this study. We excluded the patients with nasal tumor, nasal polyps, septal deviation, external ear and middle ear diseases, history of nasal sinus surgery, skull base surgery, orbital surgery, ear surgery, any surgery with contraindicating to blow the nose.

### Randomization

The randomization list was computer-generated by a statistician based on the block randomization method with randomly selected block sizes. The allocation assignment was sealed in opaque, sequentially numbered envelopes. If the patient is eligible for the trial, the envelope will be opened by a research assistant.

### Blinding

This study was single-blind. The attending physicians and evaluators were blinded from the allocation.

### Measurement protocol

After the patients agree to enroll in this study, the baseline data, nasal patency score measured by the visual analogue scale score were collected along with acoustic rhinometry test and tympanometry test of the right ear and right nose. As nasal patency may vary with room acclimatization, all procedures were performed in a controlled temperature room with a temperature setting of 21 °C.

Then, the patients were randomly assigned to perform (1) the nose-blowing with pinching by pressing a finger on the left nostril to shut the left nostril off or (2) blowing without pinching (both nostrils open). There was no specific instruction on the exhalation force the patients need to produce. The patients were instructed to perform nose-blowing as their usual habit. The dynamic closing and opening of the nostril was not allowed. The facial tissue was provided to the patients to cover the nose and to remove the mucus remnant.

Within 5 mins, the nasal patency score, acoustic rhinometry, and tympanometry test results of the right ear and right nose were collected again. Any adverse events were recorded.

### Outcomes

#### Visual Analogue Scale (VAS) of nasal patency

The VAS is a unidimensional measure of symptom intensity, which has been widely used in diverse adult populations^11^. The nasal patency VAS is a continuous scale comprised of a horizontal line. The scale is anchored by “severe obstruction” (score of 0) and “no obstruction” (score of 10)^12^. There was a strong correlation between VAS of nasal patency, Nasal Obstruction Symptom Evaluation (NOSE) questionnaire, and computational fluid dynamics of nasal airflow^13^.

#### Acoustic rhinometry

Acoustic rhinometry is a diagnostic measurement of the cross-sectional area and length of the nose and the nasal cavity through acoustic reflections^14^. This tool was used to objectively measure the minimal cross-sectional area at the nasal isthmus (MCA1) and head of the inferior turbinate (MCA2)^15^.

#### Tympanometry

Tympanometry is an acoustic evaluation used to evaluate the condition of the middle ear. This tool was used to objectively measure the middle ear pressure^16^. The normal middle ear pressure ranged between + 50 to – 200 daPa (mm water)^17,18^.

#### Adverse events

The adverse events that were collected include bleeding, pain/discomfort, and headache.

### Statistical analysis

The sample size was calculated based on the expected difference in nasal patency score of 1 ± 1.2 points. With a significance level of 0.5 and power of 90 percent, the total number of specimens required was determined to be 50 patients.

Statistical analyses were performed using the SPSS version 20. Data were described as either means (for the continuous variables) or frequencies and percentages (for the categorical variables). Significant differences between groups were determined using the Student t-test or the Mann–Whitney U test for continuous variables. The chi-square test or the Fisher exact test were used to determine whether there was a significant difference between the expected frequencies and the observed frequencies. For all tests, p < 0.05 was considered statistically significant.

### Ethical considerations

This study was approved by the Khon Kaen University Ethics Committee in Human Research (HE611338) and registered in the Thai Clinical Trials Registry (registration number: TCTR20210617005, date of registration: 16 June 2021). Written informed consent to participate in this study was provided by all patients enrolled. All authors abided by the Declaration of Helsinki.

## Results

**Figure 1:**
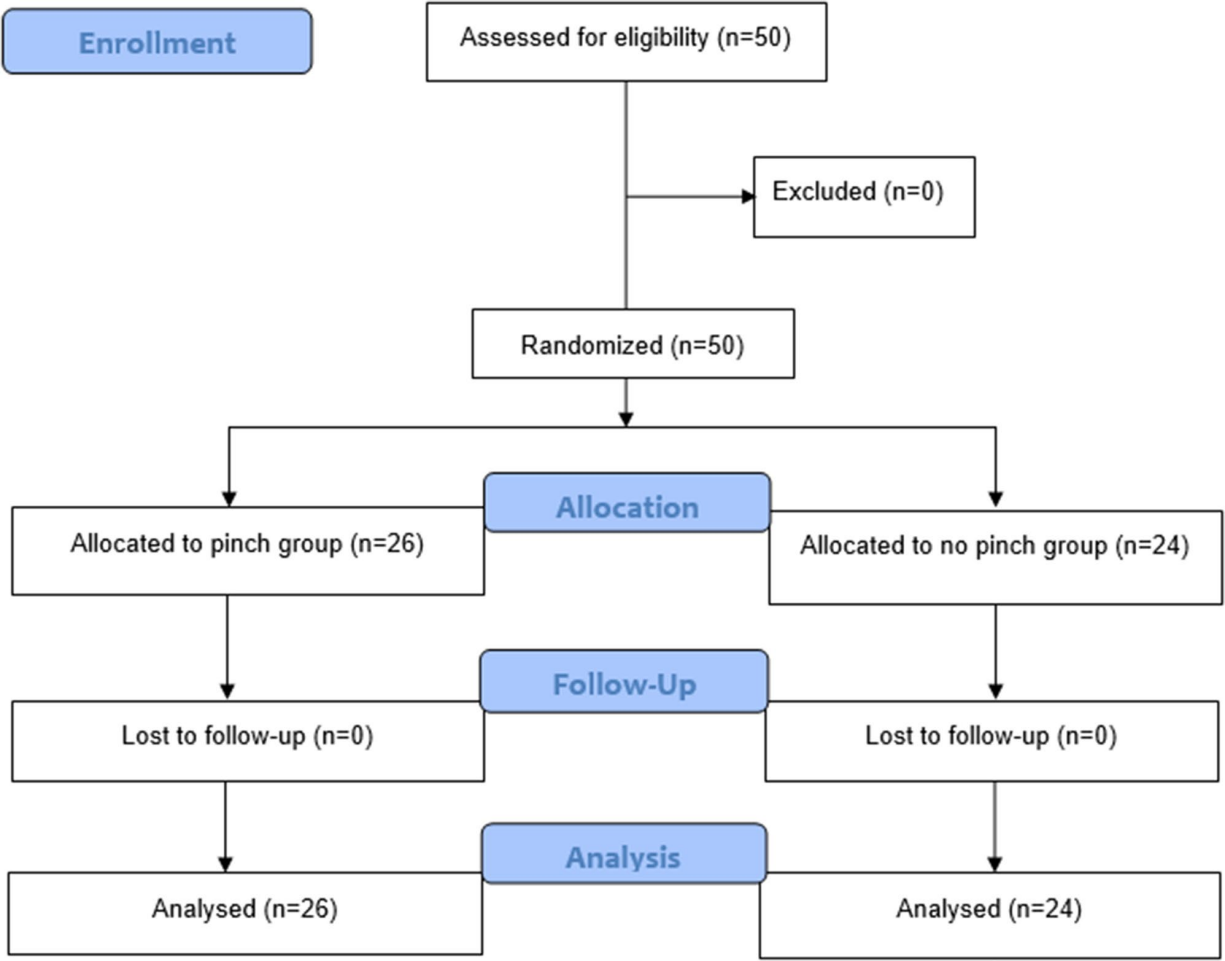
Participant flow diagram.

A total of 50 patients participated in our study. The participant’s flow diagram was shown in Fig. 1. There were 19 males and 31 females. The mean age of the patients was 42.7 years. There was no statistical difference in patients’ baseline between groups (p > 0.05) (Table 1).

**Table 1:** Baseline characteristics.

	Pinch (n = 26)	No pinch (n = 24)	p-value
**Sex**			
Male	9	10	0.263
Female	17	14	
Age (years)	44.77 ± 15.03	40.63 ± 12.71	0.300
VAS of nasal patency	5.77 ± 1.45	4.83 ± 2.04	0.066
MCA1 (cm^2^)	0.52 ± 0.14	0.48 ± 0.16	0.355
MCA2 (cm^2^)	0.60 ± 0.25	0.51 ± 0.25	0.195
Middle ear pressure (daPa)	− 28.85 ± 0.25	− 35.0 ± 34.95	0.458
**Diseases**			
Allergic rhinitis	14	17	0.216
Common cold	7	4	0.382
Chronic sinusitis	5	3	0.517

The mean VAS nasal patency score (range 0–10, higher is better) after nose-blowing in pinching group was 8.00 points and 7.13 points in no pinching group (mean difference 0.88 points, 95% CI 0.20–1.55, p = 0.012). The mean nasal minimal cross-sectional area at nasal isthmus (MCA1) was 0.53 cm^2^ in pinching group and 0.54 cm^2^ in no pinching group (mean difference − 0.003 cm^2^, 95% CI − 0.08 to 0.08, p = 0.939). The mean nasal minimal cross-sectional area at the head of the inferior turbinate (MCA2) was 0.65 cm^2^ in pinching group and 0.54 cm^2^ in no pinching group (mean difference 0.14 cm^2^, 95% CI − 0.04 to 0.27, p = 0.144) (Table 2).

**Table 2:** Nasal patency after nose-blowing.

	Pinch (n = 26)	No pinch (n = 24)	Mean difference (95% CI)	p-value
VAS of nasal patency	8.00 ± 0.94	7.13 ± 1.39	0.88 (0.20 to 1.55)	0.012*
MCA1 (cm^2^)	0.53 ± 0.14	0.54 ± 0.14	− 0.003 (− 0.08 to 0.08)	0.939
MCA2 (cm^2^)	0.65 ± 0.29	0.54 ± 0.24	0.14 (− 0.04 to 0.27)	0.144

*Significance.

After nose-blowing, the middle ear pressure in no pinching group remains relatively close to baseline (35 daPa). While in the pinching group, the middle ear pressure was increased from − 28.85 to − 17.69 daPa. However, there was no statistically significant difference in middle ear pressure between the two groups after nose-blowing (p > 0.05) (Table 3). There were no adverse events occur in both groups.

**Table 3:** Middle ear pressure after nose-blowing.

	Pinch (n = 26)	No pinch (n = 24)	Mean difference (95% CI)	p-value
Middle ear pressure (daPa)	− 17.69 ± 36.69	− 35.42 ± 37.27	17.72 (− 3.32 to 38.77)	0.097

## Discussion

Nose-blowing is a common practice to remove excess mucus from the nasal cavity in humans. However, this practice can also produce droplets and aerosol particles which contribute to the spreading of virus particles that can transmit to other persons^2,3^.

The aerosol particles expelled from the nose and mouth could be increased in vigorous respiratory activities. One study compared the airborne particles expelled during coughs versus normal exhalations. They found that 53% of patients produced aerosol particles containing viable influenza A virus during coughing, and 42% produced aerosols with the viable virus during normal exhalation. The authors conclude that although the influenza A virus was detected more often in cough aerosol particles than in exhalation aerosol particles, both respiratory activities could be important in airborne viral transmission^19^.

Furthermore, a forceful inhalation to create the intrathoracic pressure before cough, nose-blowing or sneezing can also increase the risk of viral particle deposition in the airway. One fluid dynamic study found that higher levels of exercise can increased aerosolized volume that reaches into the lower airways^20^.

We proposed that nose-blowing without nostril occlusion is a safer and gentler procedure while giving acceptable mucus clearance. To our knowledge, this is the first randomized controlled trial comparing the nasal patency and middle ear pressure after nose-blowing with pinching versus without pinching.

In this study, we found that the nasal patency score (range 0–10) was higher in the patient who pinches one nostril (mean difference 0.88 points, 95% CI 0.20–1.55, p = 0.012). However, there was no statistically significant difference in mean nasal minimal cross-sectional area between-group (p > 0.05). Moreover, the middle ear pressure between the pinching and no pinching group was also no statistically significant difference (p > 0.05).

The minimal cross-sectional area using acoustic rhinometry is the gold standard to objectively evaluate the nasal patency^21^. The recent systematic review evaluated the correlation between the subjective and objective evaluation of the nasal airway found that from 16 included studies, the correlation between the outcomes found with rhinomanometry and acoustic rhinometry and an individual's subjective sensation of nasal patency remains uncertain^22^. Therefore, using both subjective and objective evaluations would provide more value than using only one type of assessment.

We already know from the previous study that blowing the nose while close or partially close the nostril can generate higher pressure in the airway^10^. Our study took a further step by subjectively and objectively evaluate the nasal patency and middle ear pressure after nose-blowing.

We suggested that blowing the nose without pinching might be safer. The patients may need to repeat this procedure until they feel that the nose was cleared. Rather than forcefully blowing the nose that increases the chance of viral spreading and gets the risk of mucosal injury.
